# Factors Affecting Dietary Improvements in Elderly Residents of Long-Term Care Institutions Receiving Domiciliary Dental Care

**DOI:** 10.3390/medicines8110062

**Published:** 2021-10-21

**Authors:** Hitomi Kikuchi, Akira Komatsuzaki, Sachie Ono, Miwa Sirono, Shiho Motoi, Asami Iguchi, Mio Susuga

**Affiliations:** 1 Department of Dental Hygiene, The Nippon Dental University College at Niigata, 1-8 Hamaura cho, Chuo-ku, Niigata 951-8580, Japan; hitomi@ngt.ndu.ac.jp (H.K.); hsjc@ngt.ndu.ac.jp (S.M.); mio@ngt.ndu.ac.jp (M.S.); 2 Department of Preventive and Community Dentistry, School of Life Dentistry at Niigata, The Nippon Dental University, 1-8 Hamaura-cho, Chuo-ku, Niigata 951-8580, Japan; sachie@ngt.ndu.ac.jp; 3 Domiciliary Dental Care Unit, The Nippon Dental University Niigata Hospital, 1-8 Hamaura-cho, Chuo-ku, Niigata 951-8580, Japan; marumiwa@ngt.ndu.ac.jp; 4 Department of Dental Anesthesiology, The Nippon Dental University School of Life Dentistry at Niigata, Chuo-ku, Niigata 951-8580, Japan; asami@ngt.ndu.ac.jp

**Keywords:** mouth rehabilitation, elderly residents, denture wearing, diet type, activities of daily living

## Abstract

**Background:** Oral disabilities occur due to tooth loss. This study aimed to investigate oral and systemic factors related to diet in elderly residents receiving domiciliary dental care. **Methods:** The subjects were 74 consenting residents. Survey items included whether subjects could eat independently and diet type. Subjects were examined by the dentist for the number of teeth, occlusal support index, and wearing dentures. Contingency table analysis was performed to determine what levels of decline in general and oral functions led to difficulties eating a normal diet. **Results:** There was a significant difference in the mean number of activities of daily living (ADL) requiring assistance evident between subjects eating a normal diet and those eating fluid boiled rice (*p* < 0.01). A comparison of occlusal support and diet type showed that most subjects who ate a soft diet or gruel had no occlusal support. Almost all subjects who ate a normal diet wore dentures. However, only 38% of subjects eating a soft diet and 40% of those eating gruel did wear dentures; both group differences were significant (*p* < 0.01). **Conclusions:** Future studies need to further investigate oral factors related to the type of diet and their relationships to domiciliary dental care in older adults.

## 1. Introduction

Daily meals are regarded as one of the most important factors in maintaining the quality of life (QOL) of residents of long-term care institutions [1]. Decreases in systemic bodily functions are frequently evident in elderly people who require long-term care, and they also commonly have basic oral problems directly related to meals through decreases in eating and swallowing functions. Japanese seniors that have lost teeth typically do not eat a full range of food types, because it is difficult for them to obtain optimal dental care [2,3]. Long-term care services have been provided at a certain level since the introduction of long-term care insurance in Japan in 2000, but daily meals have been reported to exert a major effect on improving QOL for patients requiring long-term care. In light of the current large number of edentulous individuals in Japan [4], improving dietary lifestyle in old age as much as possible is clearly important.

Wearing dentures is necessary, not to acquire stabilized occlusion but rather to improve masticatory function. From the perspective of rehabilitation, a deteriorated oral state is a complication of major concern. Locker et al. [5] found a decrease in QOL in adult patients with a reported need for dentures (full or partial). Oral health is a significant component of the general QOL of people, with important implications for their health status [6].

Given the importance of oral care, the relationship between oral debris and soft diet, in particular, should be the subject of further investigation. Dental and denture plaque can cause or aggravate many oral diseases, such as caries (especially root caries) [7,8], periodontitis [9], oral candidiasis [10,11], denture stomatitis [12,13], and halitosis [14]. Dental plaque may also serve as a reservoir for pathogens as significant causes of pneumonia [15] and other systemic diseases [16]. A soft diet is a major factor in increasing oral debris. The importance of oral cleaning in older adults has not yet been sufficiently emphasized.

However, few reports have evaluated the effects of daily meals on health status. The associations between diet and general and oral conditions of elderly patients requiring long-term care residing in institutions that possessed kitchen facilities and provided standard meals were therefore investigated, with the aim of elucidating oral and systemic factors related to diet and domiciliary dental care [17,18,19,20].

The purpose of this study was to investigate oral and systemic factors related to diet in elderly residents receiving domiciliary dental care.

## 2. Materials and Methods

### 2.1. Participants 

This study was carried out between 2011 and 2017 in three long-term care institutions in an urban area of Japan. It was suspended for 3 years during that time due to the Great East Japan Earthquake that occurred in 2011. Dental care had been provided at these institutions since before the start of the study, and residents were provided food according to appropriate dietary standards.

All subjects gave their informed consent for inclusion before they participated in the study. The study was conducted in accordance with the Declaration of Helsinki, and the protocol was approved by the Ethics Committee of the School of Life Dentistry at Niigata, the Nippon Dental University School of Life Dentistry at Niigata (Approval No. ECNG-H-84).

The subjects were 74 residents (19 men, 55 women; mean age, 82.4 ± 8.2 years) of the institutions who consented to participate prior to enrolment and who received dental treatment at their institution of residence during the study period. Residents receiving parenteral or enteral nutrition who were unable to ingest food by mouth were excluded from the study.

### 2.2. Methods

Answers about dietary status were obtained by a questionnaire containing multiple-choice questions concerning whether the subjects could eat independently, which eating utensils they used, and the type of diet texture provided. In this study, diet texture was classified into three categories: normal diet, soft diet (soft boiled rice), and fluid boiled rice (rice gruel). Diet texture was defined as a cooking water/rice ratio of <1 for normal diet, 1.0–1.3 for soft diet, and >1.3 for fluid boiled rice. Cooking was carried out by an exclusive nutritionist.

Two dentists performed oral examinations by visual inspection, and the Eichner index (location of occlusal support zones) and using dentures were also investigated. Adhesion of debris in the oral cavity was used to assess the necessity for care. Dentures were always fitted if possible, unless residents were unable to chew due to poor general condition or they refused to wear them.

All examinations, including pre-calibrations, were performed at the long-term care institutions. The survey items are shown in Table 1.

Comparisons between diet and both general and oral conditions were summarized in a contingency table with the type of diet (normal, soft, or rice gruel) as the axis to determine which levels of decline in systemic and oral functions led to difficulties eating a normal diet and to more subjects eating a soft diet or rice gruel.

### 2.3. Statistical Analysis

Microsoft Excel (Microsoft Japan, Tokyo, Japan) statistics were used for tabulation and statistical analysis, with χ^2^ tests (for ratio difference) and *t*-tests (for mean difference) used to test for significance.

Data were collected on paper forms by nursing home staff and entered using SPSS statistical software (Dr. SPSS Package, IBM, New York, NY, USA). Potential risk factors for meal intake were assessed by incidence odds ratios (ORs) using logistic regression.

## 3. Results

### 3.1. Dietary Status

The dietary status and independent eating status are shown in Table 2. Only 20 (27%) subjects were unable to eat independently, with the type of diet being normal diet for 41 (55.4%) subjects, soft diet for 19 (25.7%), and rice gruel for 14 (18.9%). Few subjects used only chopsticks as eating utensils, with most also using multiple utensils.

### 3.2. General and Oral Conditions

Table 3 shows the general and oral conditions of the subjects. In terms of current symptoms, 31 (41.9%) subjects had a cerebrovascular disorder, and 61 (82.4%) had hypertension. The current health status was reported as good or somewhat good in most cases. The level of independence in activities of daily living (ADL) [21] was better for all subjects capable of sitting up. The mean number of ADL requiring assistance was 3.8. In terms of oral condition, 39 (52.7%) subjects were edentulous. The number of remaining teeth was only about half that found in subjects of the same age in the 2011 Survey of Dental Diseases [22].

### 3.3. Comparison of Diet Status and General Condition

These results, indicating the relevance of the type of diet with the general condition, oral condition, and use of dentures, showed that requiring assistance with ADL represented the point at which some subjects started eating a soft diet or gruel. Eating softer food types was associated with a gradual increase in the mean number of ADL requiring assistance (Table 4), and a significant difference in the mean number of ADL requiring assistance was evident between a normal diet and gruel (*p* < 0.01).

### 3.4. Comparison of Diet Status and Oral Condition

A comparison of occlusal support (Table 5) with the type of diet showed that most subjects eating a soft diet or gruel had no occlusal support. In terms of denture use, although many subjects who ate a normal diet wore dentures, 50% of subjects eating a soft diet and 40.9% of those eating gruel also wore dentures; this difference was significant (*p* < 0.01). Conversely, patients who ate a soft diet or gruel did so despite wearing dentures.

Overall, 53 (71.6%) subjects were classified as occlusal support class B4-C3 (no occlusal contacts), and 22 (29.7%) subjects were not using dentures.

In this study, adhesion of unhygienic matter was evident in 31 (41.9%) subjects, who were eating mainly soft diet or gruel. Adhesion of unhygienic matter tended to occur more commonly in patients eating highly liquid gruel.

### 3.5. Results of Logistic Regression Analyses for Meal Intake

Table 6 shows the results of logistic regression analyses for meal intake. Soft diet and gruel were significantly associated with denture wearing (OR = 5.38; 95%C.I. 1.14–25.28).

## 4. Discussion

One noteworthy finding of this study was that, based on a comparison of the number of remaining teeth and occlusal support with denture wearing, individuals who should be wearing dentures were not wearing them and were eating a soft diet or gruel. In contrast, individuals who did wear dentures were eating a normal diet, suggesting the possibility that some of the 65% of subjects who had not been fitted with dentures might be able to eat a normal diet if they were to wear them [23,24,25]. Our university has already been focusing efforts on domiciliary dental care, but an adequate system for providing this type of care has yet to be developed.

Elderly people now make up over 20% of the Japanese population, and this proportion is continuing to increase at a rapid pace. Oral and systemic functions of elderly adults need to be maintained to enable them to eat a normal diet to improve their QOL. The capacity of older adults to consume a healthy diet is an important contributor to their overall health status. Tooth loss and edentulous status are associated with lower overall dietary quality [26,27,28,29,30,31]. Education on dental health care and its improvement are therefore important. In terms of the relationship between oral debris and type of diet, although cleaning may have been performed prior to examination, adhesion of unhygienic matter was evident in one-third of all subjects in the present study, more than half of whom were eating a soft diet or gruel. It is clear that dietary intake and chewing ability are closely related to the oral environment [32].

Some limitations to this investigation need to be considered. The assessments were cross-sectional, so how participants may have eaten in the past and whether current patterns represent a departure from eating practices were not known. Whereas the analysis accounted for socio-demographic variables, other characteristics that were not measured may be associated with denture status. For example, no measure of denture quality was available, and diet type differences may have been associated with particular denture groups. As noted, the sample size for comparisons of occlusal contacts and denture quality was limited, and future research should consider these differences in a larger cohort.

In the present study, high dental care dependency did not result in a higher risk of poor oral and general health. The authors previously clarified the relationship between outpatient visits and oral symptoms in the elderly from the results of a Japanese national statistics database [33,34]. Chewing disorders should not be overlooked in efforts to prevent systemic disorders.

Although all subjects in the present study who were eating a normal diet wore dentures, the rate of denture wearing among those eating a soft diet or gruel was low, indicating a high need for dental treatment. These findings in Japanese elderly people indicate the need for future domiciliary dental care and investigations of factors, such as general condition, oral condition, and denture wearing, related to the type of diet among elderly individuals requiring long-term care to improve their diet and enhance oral health [35]. The number of remaining teeth in the subjects was about half of the average value for Japanese people [22], and it is possible that the study population had many dental diseases.

## 5. Conclusions

Oral and systemic factors related to the diet type were examined in elderly people. The impact of using dentures on diet is significant. Domiciliary dental care to improve the QOL of elderly people appears to be important. By emphasizing occlusal contact and denture wearing for improving oral function, dentists could contribute to enhancing the dietary life of elderly people. Further study of the improvement of oral health of individuals requiring nursing care who have yet to be fitted with dentures is necessary.

## Figures and Tables

**Table 1:** Survey items.

(1) Questionnaire survey (completion by nurses requested) Dietary status Can the subject eat independently? (independent, requires assistance) Eating utensils used (chopsticks, spoon, fork, other) Type of diet (normal diet, soft diet, gruel, tube feeding, other) General condition Current symptoms Level of assistance required Activities of daily living (ADL: basic seven standard actions) (2) Oral examination Teeth, occlusal support (Eichner index) Whether or not dentures are worn Plaque adhesion

**Table 2:** Dietary status.

Characteristic	Number (%)
Type of diet:	
Normal diet	41 (55.4)
Soft diet	19 (25.7)
Gruel	14 (18.9)
Independent eating:	
Able to eat independently	54 (73.0)
Unable to eat independently	20 (27.0)
Eating utensils:	
Chopsticks only	9 (12.2)
Chopsticks and spoon	40 (54.1)
Spoon	25 (33.7)
Total	74 (100.0)

**Table 3:** General and oral condition.

Survey Item	Number (%)
Current symptoms	
Hypertension	61 (82.4)
Cerebrovascular disorder	31 (41.9)
Dementia	26 (35.1)
Diabetes	21 (28.4)
Health status	
Good	47 (63.5)
Somewhat good	20 (27.0)
Poor	7 (9.5)
Number of edentulous individuals	39 (52.7)
Survey item (range)	Average ± SD
Number of ADL requiring assistance (0–7) *	3.8 ± 2.0
Number of remaining teeth (0–23)	6.1 ± 7.8

* ADL: activities of daily living (number of items requiring assistance), SD: standard deviation.

**Table 4:** Comparison of diet status and general condition.

Number of ADL Requiring Assistance	Normal Diet	Soft Diet	Gruel	Total
0–3	26 (74.3)	6 (17.1)	3 (8.6)	35 (100.0)
4, 5	11 (47.8)	9 (39.1)	3 (13.0)	23 (100.0)
6, 7	4 (25.0)	4 (25.0)	8 (50.0)	16 (100.0)
Total	41 (55.4)	19 (25.7)	14 (18.9)	74 (100.0)
Average of ADL	Normal diet	Soft diet	Gruel	
Average ± SD	2.9 ± 1.8 ^a,b^	4.1 ± 2.9 ^b^	5.5 ± 1.8 ^a^	

Identical lowercase letters indicate significant differences, *t*-test; ^a^: *p* < 0.01, ^b^: *p* < 0.05). ADL: activities of daily living, SD: standard deviation.

**Table 5:** Comparison of type of diet and occlusal support zones, denture wearing.

Oral Condition	Normal Diet	Soft Diet	Gruel	Total	χ^2^ Test
Eichner index *					
A3-B3 (Few occlusal supports)	14 (66.7)	4 (19.0)	3 (14.3)	21 (100.0)	
B4-C3 (No occlusal support)	27 (50.9)	15 (28.3)	11 (20.8)	53 (100.0)	
Dentures					
Dentures worn	39 (75.0)	8 (15.4)	5 (9.6)	52 (100.0)	**
Dentures not worn	2 (9.1)	11 (50.0)	9 (40.9)	22 (100.0)	
Plaque					
No adhesion	32 (74.4)	8 (18.6)	3 (7.0)	43 (100.0)	**
Adhesion	9 (29.0)	11 (35.5)	11 (35.5)	31 (100.0)	
Total	41 (55.4)	19 (25.7)	14 (18.9)	74 (100.0)	

* Eichner index: occlusal support index (χ^2^ test; **: *p* < 0.01).

**Table 6:** Results of logistic regression analyses for meal intake.

Explanatory Variable	OR (95%C.I.)
Dentures not worn (ref: worn)	5.38 (1.14–25.28) *
Number of ADL requiring assistance (ref: 0–3)	3.22 (0.70–15.10)
Plaque adhesion (ref: not adhesion)	1.43 (0.78–7.08)
Dependent variable: meal intake (ref: normal diet)	

Logistic Models adjusted for sex, age (*: *p* < 0.05). OR: odds ratio, C.I.: confidence interval, ADL: activities of daily living.
